# Small Molecule Activators of the Mitochondrial Protease ClpP Induce Senescence in Triple-Negative Breast Cancer Cells and Sensitize Cells to the Bcl-2 Inhibitor Venetoclax

**DOI:** 10.21203/rs.3.rs-7682325/v1

**Published:** 2025-11-19

**Authors:** Sabrina C. D. Daglish, Owen G. Canterbury, Paul R. Graves, Sarah A. Carter, Sydney M. Beese, Maggie A. Hynek, Brandon L. Mouery, Andrei J. Mistreanu, Aadra P. Bhatt, Nestor Tellez, Mitchell T. Butler, Lucas J. Aponte-Collazo, Emily M. J. Fennell, Laura E. Herring, Scott Lyons, C. Allie Mills, Hani Ashamalla, Edwin J. Iwanowicz, John P. Morris, James E. Bear, Yoshimi Endo Greer, Stanley Lipkowitz, Lee M. Graves

**Affiliations:** 1: Department of Pharmacology and Lineberger Comprehensive Cancer Center, University of North Carolina at Chapel Hill, Chapel Hill, NC, United States.; 2: Department of Radiation Oncology, New York Presbyterian Brooklyn Methodist Hospital, Brooklyn, New York, United States.; 3: Lineberger Comprehensive Cancer Center, Center for Gastrointestinal Biology and Disease, and the Department of Cell Biology and Physiology, Chapel Hill, NC, United States.; 4: Department of Cell Biology and Physiology, University of North Carolina at Chapel Hill, Chapel Hill, NC, United States.; 5: Michael Hooker Proteomics Core Facility, University of North Carolina at Chapel Hill, Chapel Hill, NC, United States.; 6: Madera Therapeutics, LLC, Cary, NC, United States.; 7: Women’s Malignancies Branch, National Cancer Institute, National Institutes of Health, Bethesda, MD, United States.

## Abstract

ONC201 is a first-in-class, FDA approved small molecule activator of the mitochondrial ATP-dependent caseinolytic peptidase P (ClpP). This and other related small molecules referred to as ClpP agonists, exert antiproliferative effects in several cancer cell types. We report that ONC201 and highly potent second generation ClpP agonists (TR-57, TR-107), promote induction of senescence in triple-negative breast cancer (TNBC) cell lines. Senescence was determined by increased β-galactosidase activity, downregulation of phosphorylated Rb, c-Myc (Myc), and lamin B1, upregulation of senescent-associated secretory phenotype (SASP), and extended cell proliferation assays. These responses were not observed in ClpP knockout cell lines, demonstrating ClpP-dependence. Proteomics analyses identified multiple events related to the development of senescence including cell cycle arrest and mitochondrial dysfunction. Flow cytometry confirmed an S-phase arrest; DNA damage was detected by Comet assay, 53BP1, phospho-S*Q, and γH2A.X immunostaining. In parallel with this, activation of the ATM pathway and phosphorylation of Chk2 was observed. We determined that ClpP agonist-induced senescence was irreversible in both *in vitro* and *in vivo* studies. Following TR-57 treatment and drug washout, cells remained growth arrested which coincided with the loss of Myc protein. By contrast, cells treated with the cell cycle inhibitor and senescence inducer, abemaciclib rapidly regained p-Rb and Myc expression and cell proliferation following washout. This response was reproduced *in vivo* wherein senescent 4T1-Luc cells did not develop tumors following injection into mice. Finally, the combination of a ClpP agonist with a known senolytic (venetoclax), synergistically increased the amount of cell death observed. Combining a ClpP agonist with a PARP inhibitor (olaparib) produced an additive effect. In summary, we show that ClpP activators stably induce an irreversible senescence in a ClpP-dependent manner that synergizes with venetoclax in TNBC cells.

## Introduction:

Triple-negative breast cancer (TNBC) is the most aggressive subtype of breast cancer with a significantly lower survival rate than other subtypes (1–3). Since TNBC cells lack estrogen and progesterone hormone receptors or enrichment of the receptor tyrosine kinase HER2, targeted therapeutics like tamoxifen and trastuzumab are largely ineffective (2,4). Current treatments for TNBC patients are limited to surgical intervention and traditional chemotherapies, and third-line therapies include immunotherapy, PARP inhibitors, and antibody drug conjugates (5–7). While there are potential targeted therapies in clinical trials for TNBC, none of them have reached FDA approval, therefore there is an unmet need for improved therapies for TNBC patients (7,8)

ONC201 was the first imipridone molecule to show strong anti-cancer properties. ONC201(Dordaviprone) is in multiple clinical trials for a variety of aggressive cancers including blood, breast, and endometrial cancers among others and recently received FDA approval for the treatment of diffuse midline glioma (9–14). Although initially believed to be a TRAIL inducer and/or dopamine receptor D2 antagonist, Greer, et al. (2018), determined that ONC201 induces mitochondrial dysfunction (15). Further studies identified ONC201 and related molecules as highly selective activators of the mitochondrial protease ClpP (referred to as ClpP agonists hereafter). This includes ONC206, ONC212, the highly potent TR compounds, and related small molecules (15–21). Of these, the TR compounds exemplified by TR-57, TR-107 and others (19,22–25) (Madera Therapeutics), are the most potent and selective ClpP agonists known (19,22–25). TR-57 and TR-107 were primarily evaluated in this study because they have been well characterized as selective ClpP activators with anti-cancer properties in TNBC (19,23,24,26).

ONC201 and the TR compounds directly bind and activate ClpP, a mitochondrial matrix protease (19,21), part of the multimeric ClpXP protein complex. ClpXP includes the ATP-dependent unfoldase ClpX and the serine protease subunit ClpP. ClpP regulates mitochondrial protein homeostasis (27) and has been implicated in degrading proteins as part of the mitochondrial unfolded protein response (28–30). Small molecule ClpP agonists bind to the hydrophobic H site cleft of the tetradecameric form of ClpP, preventing ClpX binding and inducing conformational change and activation of ClpP (23,27). ClpP activation induces degradation of multiple mitochondrial proteins leading to increased mitochondrial stress responses (19), inhibition of mitochondrial transcription (25), disruption of TCA cycle metabolism, OXPHOS, (22,24,31), and loss of mitochondrial DNA (15). These mitochondrial-related events induce the integrated stress response, inhibit protein synthesis, and arrest cancer cell proliferation (19,22,32). While many of the direct results of ClpP activation have been identified, the mechanisms that inhibit cancer cell proliferation are only partially understood. Moreover, various cancer types respond differently to ClpP agonists (19,33–36), illustrating the need to better understand the specific effects of these anti-cancer compounds.

Cellular senescence is characterized by permanent cell cycle arrest, DNA damage, decreased expression of pro-apoptotic proteins, resistance to apoptosis, and increased secretion of cytokines and proteins known as the senescence-associated secretory phenotype (SASP) (37–40). DNA damage, expression of anti- or pro-apoptotic proteins, and the SASP components vary based on the senescence inducer and tissue type (39,41). For example, cancer drugs such as abemaciclib and palbociclib induce senescence through inhibition of the cell cycle without inducing DNA damage (42–45). Increased SASP is a common feature of senescent cells, but the phenotype varies in replicative, DNA damage-induced, oncogene-induced, and mitochondrial dysfunction-induced senescence (39,43,46–48).

In this study, we examined the effects of highly selective ClpP agonists on growth inhibition and senescence of TNBC cell models. We observed that ClpP activation induces DNA damage, cell cycle arrest, SASP, and senescence in response to ClpP agonist treatment. Compared to other senescence inducers, ClpP agonist-induced senescence was not reversible. Consistent with increased senescence, the combination of ClpP agonists with an established senolytic (venetoclax) resulted in a synergistic increase in cell death. Thus, these studies could have important implications for the use of ClpP agonists in the treatment of TNBC.

## Methods:

### Chemicals

The TR-57 and TR-107 compounds were supplied by Madera Therapeutics, LLC. Other compounds were obtained as follows: ONC201 (SelleckChem, S5716), Abemaciclib (SelleckChem, S5716), Irinotecan (MedChemExpress, HY-16562), Venetoclax (MedChemExpress, HY-15531), Olaparib (SelleckChem, S1060), and KU-60019 (MedChemExpress, HY12061).

### Cell Culture

The human TNBC cell line, SUM159, was obtained from Dr. Gary Johnson at UNC-CH. MDA-MB-231, including ClpP knockout (KO) cells, were generous gifts from Yoshimi Greer and Stan Lipkowitz at the NCI. SUM159 cells were cultured in Dulbecco’s modified Eagle’s medium: Nutrient Mixture F-12 supplemented with 5% fetal bovine serum, 5μg/mL insulin, 1μg/mL hydrocortisone, and 1% antibiotic−antimycotic (Gibco, 15240062). MDA-MB-231 cells were cultured in RPMI 1640 media supplemented with 10% FBS and 1% antibiotic−antimycotic. 4T1-Luciferase cells were cultured in RPMI 1640 supplemented with 10% FBS, 1% antibiotic-antimycotic, and 4μg/mL Blasticidin (InvivoGen, BLL-44–07). Cell lines were incubated at 37°C and 5% CO_2_ and periodically tested for mycoplasma.

### Generation of ClpP null cells using CRISPRi

To generate sgRNA for CRISPRi, primer pairs for each individual sgRNA were first annealed and later ligated to a digested VDB783 vector (50ng/μL). Primer sequences are listed in Table 1. Each ligation product was then transformed into DH5α by mixing 3μL of DNA into 25μL of competent cells. Cells/DNA mixture was incubated on ice for 30 min and heat shocked at 42°C for 45 seconds. Bacteria were spun at 10 000xg for 1 min at room temperature on a tabletop centrifuge. The resulting bacterial pellet was resuspended in 30μL of LB, spread on an LB-Amp plate and incubated at 37°C overnight. Colony PCR was performed to check for positive clones of each sgRNA. Single positive clones were grown in 5mL of LB supplemented with Ampicillin at 37°C overnight. Cultures were miniprepped the next day using a QIAprep Spin Miniprep Kit (Qiagen) according to the manufacturer’s protocol. Lentiviruses were produced in HEK293T cells. Transfection and clonal isolation of the CRISPR null mammalian cells was done as previously described in SUM159 cells (22).

### Generation of 4T1-luciferase (4T1-Luc) cells

4T1 cells were purchased from the Tissue Culture Facility at UNC-Chapel Hill and maintained in RPMI-1640 containing 1% penicillin-streptomycin, 10% heat-inactivated FBS, HEPES (pH 7.0), 1mM sodium pyruvate, and 0.075% sodium bicarbonate. Cells were transduced with pTK1261 (LV-CMV-FLuc-IRES-GFP/BSD) wherein the firefly luciferase and the fusion GFP/blasticidin marker gene were expressed under the control of a CMV promoter; cells were selected with blasticidin (4μg/ml) and were clonally selected for high luciferase expression (BMG Clariostar Plate reader, Cary, NC, USA). Identity of the parental and luciferase-expressing clones were verified by Short Tandem Repeat Analysis (Powerplex 16HS, Promega), and cells were routinely monitored every two weeks for mycoplasma Mycostrip detection kit (Invivogen). Prior to *in vivo* experiments, growth rates of parental and luciferase-expressing clones were confirmed to be similar (CellTiter Aqueous One MTS assay, Promega).

### Senescence-associated β-galactosidase (β-gal) staining

SUM159 and MDA-MB-231 cells were stained for β-gal activity using an adjusted protocol from Dimri et al., 1995 (49). After treatment with senescence inducing compounds, the cells were washed twice with Dulbecco’s phosphate buffered saline (DPBS, Gibco, 14190–144) and fixed with fixing solution (2% formaldehyde, 0.2% glutaraldehyde in DPBS) for 3–5 min. After fixation, cells were washed gently 3 times with DPBS. Next, the cells were stained with staining solution (1mg/mL X-gal (Roche, XGAL-RO), 40mM citric acid/sodium phosphate buffer (pH 6.0), 5mM potassium ferrocyanide, 5mM potassium ferricyanide, 150mM sodium chloride, and 2mM magnesium chloride). Plates were covered with parafilm and incubated for 13 and 16 h for SUM159 and MDA-MB-231 respectively at 37°C without CO_2_. After incubation, the cells were washed with DPBS and imaged in DPBS on the Olympus IX70 inverted microscope (Tokyo, Japan) at 10x magnification. β-gal positive cells were counted on ImageJ with >100 cells/condition.

### Dose-Response and Drug Washout Proliferation Assays

#### Dose-Response:

Proliferation assay was performed as described previously in Daglish et al. (2023) with modifications based on treatment duration (25). Briefly, SUM159 (200 cells/well) or MDA-MB-231 (500 cells/well) were plated in a black with clear bottom 96-well plate and allowed to adhere overnight. Cells were treated with varying concentrations of TR-57 and incubated at 37°C for 3 days or 30 days. Staining, imaging, and quantification steps are described below.

#### Drug Washout:

SUM159 (1 000 cells/well), MDA-MB-231 (2 000 cells/well), or 4T1-Luc cells (500 cells/well) were seeded and allowed to adhere overnight. Cells were treated with TR-57 or controls for 24 or 48h and then drugged media was removed and replaced with regular media which remained for the duration of the experiment. To determine cell count, Hoechst 33342 (Thermo Fisher Scientific, H3570) was diluted in DPBS to 2.5μg/mL and 100μl of the stain was added to each well and plate was incubated for 20 mins at 37°C. Total cell number was determined by imaging and quantified using the Celigo Imaging Cytometer (Nexcelom, Lawrence, MA, USA).

### Immunoblotting

Cells were plated in a 6-well plate or 10 cm dish and treated with compounds as described above. Following treatment, cells were lysed with RIPA buffer, pH 7.4 [no SDS, 2mM Na(VO3)4, 10mM NaF, 0.0125μM calyculin A, and complete protease inhibitor cocktail (Roche, 11873580001)] and lysates immunoblotted as described previously (19). For secreted protein immunoblots, cell culture media was collected and 4X Laemmeli buffer was added before immunoblotting as described previously(19). Nitrocellulose membranes were first incubated in 1% fish gelatin (Sigma-Aldrich, G7041) for 1 h and then incubated with the indicated primary antibody (Table 2) in 1% fish gelatin overnight at 4°C. After incubation, the membranes were washed 3 times for 5 min with Tris-buffered saline (supplemented with 0.1% Tween-20 (TBST). Membranes were then incubated with the respective secondary antibody for 1 h at room temperature. After incubation, the membranes were washed 3 times for 5 min with TBST prior to incubation in enhanced chemiluminescence reagent (BioRad, 1705061) for 1 min and imaged using a Chemidoc MP (BioRad, Hercules, CA, USA). Images acquired were analyzed using Image Lab software (BioRad).

### RNA extraction and cDNA synthesis

Total RNA was extracted and purified using RNeasy Plus Mini Kit (Qiagen, 74136) according to the manufacturer’s protocol. RNA concentration was determined using the NanoDrop One spectrophotometer (Thermo Scientific, Waltham, MA, USA). cDNA was synthesized from reverse transcription on 2.0μg total RNA in a 20μL reaction using High-Capacity cDNA Reverse Transcription Kit (Applied Biosystems, 4368814) and T100 thermal cycler (BioRad), according to the manufacturer’s protocol.

### Quantitative real-time PCR (qRT-PCR)

The cDNA was analyzed by qRT-PCR using iTaq Universal SYBR Green Supermix (BioRad, 1725121) on an Applied Biosystems 7500 Fast Real-Time PCR System. For each reaction, 1 μL of cDNA was mixed with 12.5 μl of 2 x SYBR mix, 8 μL of nuclease-free water, 1.75 μl of Forward primer and 1.75 μl of Reverse primer. Expression of 18S was used to normalize expression of genes of interest. Every biological replicate was analyzed in technical duplicate. Primer targets and sequences are listed in Table 3.

### Proteomics

#### Sample Preparation for Proteomics:

MDA-MB-231 cells were plated in 10cm plates at a density of 2 000 000 cells per plate and allowed to adhere overnight. Plates were then treated with either 0.1% DMSO or 150nM TR57 for 48 h. Cells were washed thrice with 5mL of ice-cold DPBS. Harvesting was completed by mechanical scraping (Corning, 3008) on ice in DPBS, centrifugation at 3000 rpm for 3 min, and resuspension of the pellet in 100μl 8M urea lysis buffer (8M urea, 50 mM Tris (pH 7.4), 2.5 mM Na3VO4, 1 mM NaF, 1X protease inhibitor cocktail (Sigma, 11836170001), 12.5nM Calyculin A). Samples were incubated on ice for 20 min and then clarified by centrifugation at 15000 rpm for 10 min. Protein concentration was quantified by Bradford assay (BioRad, 5000006) and normalized to 1μg/μl using molecular biology grade water. Samples were then flash frozen in liquid nitrogen and submitted on dry ice to the UNC Metabolomics and Proteomics Core. Cell lysates (n=12 per group) were subjected to S-trap digestion, as previously described (50). Approximately 35 μg of sample were reduced with 20 mM dithiothreitol (Pierce) for 10 min at 56 °C, and alkylated with 40 mM iodoacetamide (Pierce) for 30 min at room temperature. Samples were then loaded onto S-trap Micro columns (Protifi) according to manufacturer’s recommended protocol. Samples were subjected to on-column digestion using trypsin (Promega) for 1 h at 47 °C at a 1:10 enzyme:protein ratio. Eluates were dried via vacuum centrifugation (Labconco, Kansas City, MO, USA) and peptide concentration was quantified using BCA Fluorometric Peptide Assay (Pierce). Peptide clean-up was performed on Evotips as recommended by the manufacturer.

#### LC/MS/MS Analysis:

Each sample was analyzed by LC-MS/MS using an Evosep One coupled to a Fusion Lumos mass spectrometer (Thermo Scientific). Peptides were separated with the 15 SPD Extended Method, a standardized 88-min method using a ReproSil-Pur C18 column (15 cm × 150 μm, 1.9 μm beads, Evosep). The mobile phases for separation were 0.1% formic acid in water for buffer A and 0.1% FA in acetonitrile for buffer B. The Lumos was operated in Data-Independent Acquisition (DIA) mode. A full MS scan (m/z 35–1200 m/z) was collected; resolution set to 120 000 with a default charge state of 2, max injection time of 45 ms and automatic gain control (AGC) target of 250%. Following full MS scan, product ion scan was collected at a resolution of 30 000, with higher collision dissociation (HCD) set to 30, AGC target set to 2000%, maximum injection time to set to 54 ms, 31 m/z precursor isolation windows.

#### Data Analysis:

Raw data were analyzed using directDIA within Spectronaut (v18, Biognosys). Data were searched against the reviewed human proteome database from Uniprot (downloaded January 2024; 20,441 protein sequences); appended with a common contaminants database (MaxQuant; 245 protein sequences). The following settings were used: enzyme specificity set to trypsin, up to two missed cleavages allowed, cysteine carbamidomethylation set as a fixed modification, methionine oxidation and N-terminal acetylation set as variable modifications. A false discovery rate (FDR) of 1% was used to filter all data; and proteins identified by only one peptide were excluded from the results. Within Spectronaut, log2 fold change ratios of each pairwise comparison was calculated, along with a q-value (FDR-corrected p-value from Student’s t-test). R (version 4.4.1) was used for further analysis and figure generation (PCA plot). VolcanoseR was utilized for generation of volcano plots (51). GO pathway enrichment analysis was performed using the clusterProfiler package (52). The proteomics datasets generated and analyzed in this study are available in the Proteomics Identification Database (PRIDE) repository under project identifier PXD067842.

### Apoptotic Antibody Array

The Proteome Profiler Apoptosis Array kits were purchased from R&D Biosystems (Cat#ARY009). Cells were plated in 10 cm dishes (1 000 000 cells/plate) and treated with compounds as described above. Cells were manually scraped and pelleted, then lysed with provided lysis buffer as detailed in kit. After lysis and clarification by centrifugation (10 mins at 15 000 rpm), protein concentration was determined by Bradford and 400 ug was added to array membrane. After cell lysis and protein concentration determination, the kit instructions were followed for blotting and imaging. Array membranes were imaged using a Chemidoc MP (BioRad) and images were quantified using Fiji software (ImageJ).

### Immunofluorescence

SUM159 cells were cultured at 5×10^4^ cells per well in an 8 well chambered slide (ibidi, 80806). After cells were treated for indicated durations, the cells were fixed with 4% formaldehyde in DPBS for 10 min at 37°C. Then the slide was washed thrice for 5 min with DPBS on the orbital shaker. The cells were permeabilized with permeabilization solution (5% bovine serum albumin (BSA), 100mM glycine, 5% donkey serum (Sigma, D9663), 2% Triton X-100) for 15 min at room temperature on the shaker. Next the cells were incubated in blocking solution (5% BSA, 100 mM glycine, 5% donkey serum) for 30 min at room temperature on the shaker. Primary antibodies were added at indicated dilutions (see Table 2) in 5% BSA and 100mM glycine solution and cells were incubated and shaken in this solution overnight at 4 °C covered from light. The next day, cells were washed thrice for 5 min with DPBS. Then the secondary antibody solution (5% BSA, 100mM glycine, indicated dilutions of secondary antibody) was added to the cells and shaken for 1 h covered from light at room temperature. Next, 2nM DAPI in DPBS solution was incubated on the cells for 10 min. The cells were washed twice for 5 min before being stored at 4 °C until ready for imaging. The slides were imaged on a Zeiss LSM700 confocal microscope using an EC Plan-Neofluar 40x/1.30 Oil DIC M27 oil objective with a 38μm optical section (Oberkochen, Germany). Images were collected at 1024 × 1024 pixels with 8 bits per pixel, using 0.2% laser power of a 5mW 405 nm laser, 10mW 488 nm laser, and 10mW 561 nm laser. Images were analyzed using custom Python scripts with at least 50 cells per condition counted. Images were imported and processed utilizing scikit-image. Nuclear segmentation was employed using skimage on DAPI channel, and foci were counted after manual thresholding in red and green channels within the nucleus areas (53). Data was exported, then graphed and statistically analyzed in Prism.

### Flow Cytometry

#### Cell Cycle:

The flow cytometry protocol was modified from Mouery, et al. (2024) (54). SUM159 and MDA-MB-231 cells were supplemented with 10 μM EdU for 30 min before being trypsinized and pelleted at 1000×g for 5 min. After a DPBS wash, the cells were fixed for 15 min at room temperature with 4% formaldehyde. Next, the cells were washed with 1% BSA in DPBS and stored in 4°C until staining. Cells were permeabilized with 1% BSA and 0.5% Triton X-100 for 15 min before EdU staining was performed. The staining solution (1mM copper (II) sulfate, 100 mM ascorbic acid, and 1 mM Alexa Fluor 647 Azide (Invitrogen, A10277) in DPBS) was added to permeabilized cells, protected from light, and incubated at room temperature for 30 min. After washing the cell pellet with 1% BSA and 0.5% Triton X-100 in DPBS, the cells were incubated overnight at 4 °C in DAPI stain (1μg/mL DAPI, 100μg/mL RNAse, 1% BSA, 0.5% Triton X-100 in DPBS). Cells were filtered in 35μm filtered test tubes (Falcon, 352235) before being imaged on the Attune NXT cytometer (New York City, NY, USA). Cell cycle data were analyzed using FCS Express.

#### Annexin V/Propidium Iodide Experiment:

Protocol was adapted from the Der Lab (UNC (55). TNBC cell lines were treated for 72h with drug combinations, then media was collected, and remaining cells were washed with DPBS then detached with trypsin/EDTA (Gibco, 15400054). Floating cells from media and trypsinized cells were combined and collected by centrifugation. After a DPBS wash and repelleting, the cells were resuspended in 100μl staining solution (5% Annexin V-AF488 (Invitrogen, A13201), 1μg/mL propidium iodide (Invitrogen, P1304MP) in Annexin V Binding Buffer (10mM HEPES (pH 7.4), 140mM NaCl, 2.5mM CaCl2)) and left to incubate in the dark at room temperature for 15 min. Next, cells were further diluted into 400μl Annexin V Binding Buffer and filtered into flow test tubes (Falcon, 352235) before being read on the Attune Nxt Cytometer. Data were analyzed utilizing Floreada.io (https://floreada.io, last accessed June 21, 2025)

### Comet Assay

A modified comet assay protocol was adapted from Fagan-Solis, et al. (2020) utilizing the CometAssay Single Cell Gel Electrophoresis Assay kit (R&D Systems, 4250–050-K) (56). Briefly, 5,000 cells were mixed with 0.5 mL of LMAgarose and plated on slides. The slides were submerged in CometAssay Lysis solution overnight at 4°C. After 1 h incubation in Alkaline Unwinding Solution (200mM NaOH, 1mM EDTA pH >13), gel electrophoresis was run on the slides with Alkaline Electrophoresis Solution (200mM NaOH, 1mM EDTA, pH >13) at 21V for 1 h at 4 °C. Slides were suspended in deionized H_2_O (diH_2_O) twice for 5 min and then with 70% ethanol for another 5 min. Samples were dried for 15 min at 37°C and then incubated with 1X SYBR Gold (Invitrogen, S11494) solution (dissolved in TE buffer (10mM Tris (pH 7.5), 1mM EDTA) for 30 min at room temperature in the dark. Slides were washed with diH_2_O before being imaged on a ZEISS Axio Vert.A1 inverted microscope at 10x magnification.

### Cell Line-Derived Xenograft Mouse Experiment:

All animal studies were carried out following approval from the UNC Institutional Animal Care and Use Committee (Protocol# 22.215). Arrive2.0 guidelines were observed, including randomization of mice to study groups and blinding investigators involved in the study. 4T1-Luc cells were treated with 150nM TR-57 for 24 or 48h, or 0.01% DMSO for 48h for controls. After treatment, cells were detached with Cell Stripper (Corning, 25–056-CI) before being counted and pelleted. Cells were resuspended at 5×10^6 cells/mL in sterile 1X DPBS and placed on ice until injection. Female BALB/c mice were injected on the left mammary fat pad with 100μl of 4T1-Luc cell suspension. A total of 8 mice per condition were tested. Body condition, weight, and tumor formation were monitored 2x/week; tumor volumes were assessed by intravital imaging (IVIS Optical Imager, Waltham, MA, USA) performed on anesthetized mice that were intraperitoneally injected with d-luciferin (75mg/kg). Study endpoints were maximum tumor volume (2000 mm^3) or declining body condition (e.g. weight loss of 20%). Maximum tumor burden was not exceeded in this study. TR-57-pretreated mice did not reach either endpoint and accordingly, the study was terminated after 55 days.

### Synergy Dose-Response and Analysis

SUM159 and MDA-MB-231 cells were plated at 1 000 and 2 000 cells/well respectively and plated in black with clear bottom 96 well plates. The Tecan D300e liquid handler (Männedorf, Switzerland) was used to treat the cells with TR-57 and venetoclax or olaparib, then cells were incubated for 72h. 100μl of 5μg/mL propidium iodide and 2.5 μg/mL of Hoechst 33342 stain in DPBS was added to each well. Cells were incubated for 20 min at 37°C. Then cells were imaged on the Celigo Imaging Cytometer and dead and total cell count was determined with the Celigo software. Percent viability was calculated and input into SynergyFinder+ (57). HSA synergy scores were exported and graphed in heatmaps using GraphPad Prism.

### Statistical Analysis

Statistical calculations for all the data, excluding proteomics, were performed using GraphPad Prism. Data are reported as the mean ± standard deviation which was performed on all datasets to determine positive and negative errors. Unpaired two-tailed student t-test, one-way, or two-way ANOVAs were used to make comparisons between groups, and p values below 0.05 at the 95% confidence level were considered to be statistically significant. The number of significance stars are based on GraphPad reporting style. When brackets are not used to compare experimental groups, the statistical star is for the indicated group compared to the DMSO control at that timepoint.

## Results:

### ClpP activation induces senescence and increases SASP

**Figure 1:**
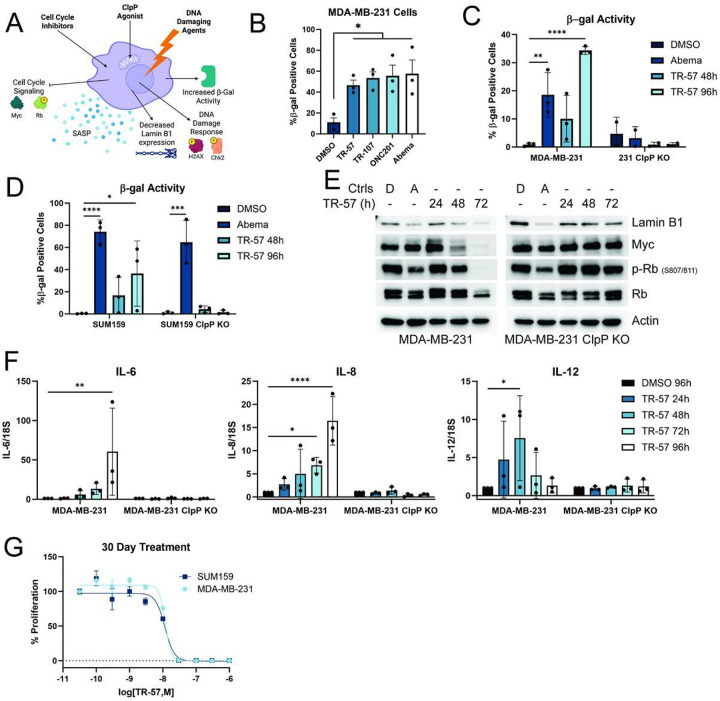
ClpP activation induces senescence and the SASP in TNBC cell lines A) Model depicting initiators of senescence and the consequences on cell markers. B) MDA-MB-231 cells were treated with 0.1% DMSO, 150nM TR-57, 100nM TR-107, 10μM ONC201, or 500nM abemaciclib for 96h. β-galactosidase (β-gal) activity was measured by colorimetric assay and quantified, N=3. C-D) MDA-MB-231 or SUM159 and respective ClpP KO cell lines were treated with 0.1% DMSO (96h), 500nM abemaciclib (48h) or 150nM TR-57 (48 or 96h). β -gal activity was measured and quantified, N=3 E) Immunoblots of MDA-MB-231 and ClpP KO cells treated with DMSO (D) for 72h, abemaciclib (A) for 48h, or TR-57 for indicated timepoints. Representative images of N=3. F) qRT-PCR determined relative expression levels of IL-6, IL-8, and IL-12 after treatment in MDA-MB-231 and ClpP KO cells, N=3. G) TNBC cell lines were treated with varying concentrations of TR-57 for 30 days. Representative of N=3. All error bars are representative of the standard deviation (SD).

We previously observed that exposure of TNBC cell lines to ClpP agonists induced cytostasis but not cell death (19,22). To further investigate this, we examined multiple events associated with cell senescence including β-galactosidase (β-gal) activity, lamin B1 expression, cell proliferation, and SASP markers as shown (Fig. 1A). Incubating MDA-MB-231 cells with three chemically different ClpP agonists (TR-57, TR-107, ONC201) resulted in significant increases in β-gal activity (Fig. 1B, and Supp. Fig. 1A). For simplicity we focused on the potent and selective ClpP activator TR-57 for many of the studies described hereafter. Abemaciclib was used as a positive control for therapy-induced senescence in most cases (44). TR-57 induced significant increases in β-gal staining after 96h treatment in both TNBC cell lines. However, TR-57 did not increase β-gal activity in the ClpP knockout (KO) cell lines (SUM159 KO or MDA-MB-231 KO), confirming ClpP dependence (Fig. 1C–D, Supp. Fig. 1B-C). Loss of lamin B1, an established marker of senescence (58), was observed after abemacicilib and TR-57 treatment of SUM159 and MDA-MB-231 cells (Fig. 1E, Supp. Fig. 1D). Furthermore, TR-57-dependent growth inhibition was confirmed by decreased retinoblastoma protein (Rb) phosphorylation and a reduction in c-Myc (Myc) protein after 48 h, whereas lamin B1, Myc, or Rb phosphorylation was unaffected in the ClpP KO cells following TR-57 treatment (Fig. 1E, Supp. Fig. 1D). Other ClpP agonists also decreased lamin B1, p-Rb, and Myc protein expression, demonstrating that these senescence markers are consistently affected by chemically different ClpP agonists (Supp. Fig 1G).

The secretion of cytokines, chemokines, MMP’s, and other proteins (GDF15) is a hallmark of senescence collectively known as the SASP (46,59,60). As determined by qRT-PCR, TR-57 incubation strongly increased IL-6, IL-8, and IL-12 mRNA in MDA-MB-231 cells whereas no significant differences were observed in the MDA-MB-231 ClpP KO cells (Fig. 1F). Western blotting for growth differentiation factor 15 (GDF15), demonstrated increased GDF15 protein levels after TR-57 treatment of SUM159 cells (Supp. Fig. 1E). Lastly, we performed long-term incubation assays to determine if ClpP agonists were stably inducing senescence. TNBC cells were cultured for 30 days with TR-57 to determine if growth arrest was permanent under these conditions. After 30 days, no increase or decrease in total cell count was observed at the higher concentrations of TR-57, suggesting that the TNBC cells were neither dying nor proliferating (Fig. 1G). Comparison of the IC_50_ values from the 30-day incubation showed that they were not significantly different from that obtained after 3-day TR-57 incubation (Supp. Fig. 1F). Taken together, these data demonstrate that the ClpP-dependent inhibition of cellular proliferation in TNBC cell models is accompanied by increased markers and evidence of stable cell senescence.

### Proteomics data identifies protein changes related to cell cycle arrest and senescence

**Figure 2:**
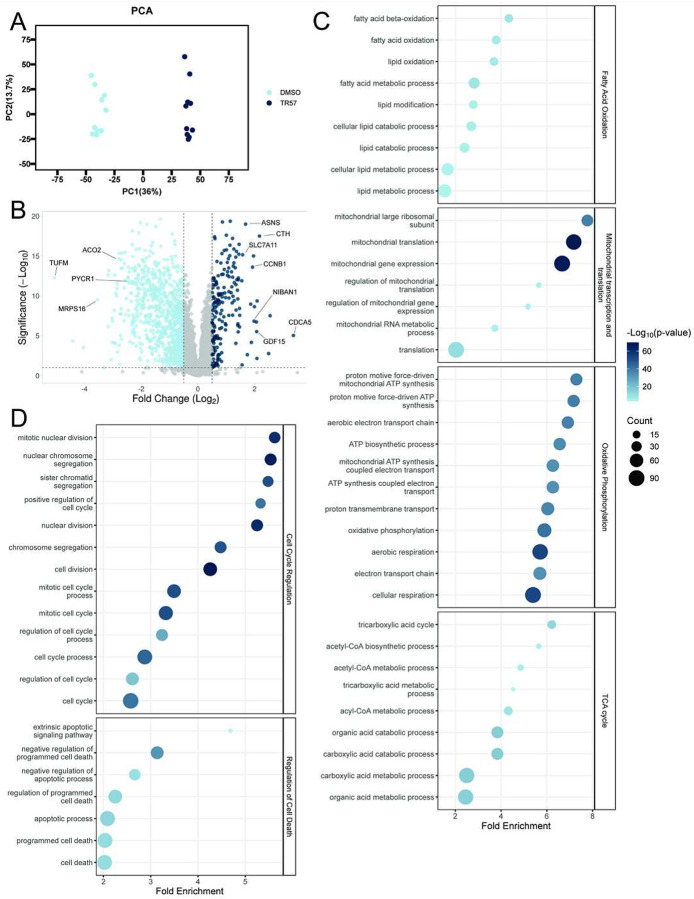
Proteomics analysis indicates TR-57 induces mitochondrial dysfunction, cell cycle arrest, and regulation of cell death MDA-MB-231 cells were treated with 0.1% DMSO or 150nM TR-57 for 48h and whole cell proteomics was performed. A) Principal component analysis (PCA) plot demonstrates distinct clustering of the DMSO and TR-57 samples. B) Volcano plot analyses show significant increases (right) and decreases (left) in protein expression after TR-57 treatment. GO pathway analysis of significantly decreased proteins (C) and increased proteins (D) after TR-57 treatment (48h).

Because our data suggested that exposure of MDA-MB-231 cells to TR-57 for 48h was sufficient to induce senescence, we performed proteomics analysis on these cells (31). Principle component analysis (PCA) demonstrated excellent clustering of the DMSO and TR-57-treated samples (Fig. 2A). Analysis of this data confirmed that TR-57 caused down-regulation of multiple mitochondrial matrix proteins (mitochondrial elongation factor Tu (TUFM), aconitate hydratase (ACO2), Pyrroline-5-carboxylate reductase 1 (PYCR1), small ribosomal subunit protein bS16m (MRPS16), others) as observed earlier (24h TR-57), albeit to a greater extent (31). Volcano plot analyses identified additional upregulated proteins indicating activation of cyclic-AMP dependent transcription factor (ATF4) and the integrated stress response (SLC7A11, asparagine synthetase (ASNS), cystathionine gamma-lyase (CTH), NIBAN1), cell cycle arrest (Sororin (CDCA5), G2/mitotic specific cyclin B1 (CCNB1)), and cell senescence (GDF15) (Fig. 2B). We next applied GO pathway enrichment analysis to evaluate the effects of TR-57 on cellular signaling processes. As expected, multiple mitochondrial functions (Tricarboxylic acid (TCA) cycle, oxidative phosphorylation) and mitochondrial-associated pathways (fatty acid oxidation, mitochondrial transcription and translation) were disrupted (Fig. 2C). Additional events related to cell cycle regulation (mitotic nuclear division, chromosome segregation) and regulation of cell death (negative regulation of cell death) were also observed (Fig. 2D).

The expression of pro- and anti-apoptotic proteins were examined using commercial antibody arrays. These results demonstrated that the pro-apoptotic proteins Bad, SMAC/Diablo, and others were downregulated in SUM159 and MDA-MB-231 cells after TR-57 treatment (Supp. Fig. 2 A-B). TR-57 also resulted in the downregulation of specific anti-apoptotic proteins, cellular inhibitor of apoptosis protein 1 (cIAP1), Survivin, and others (Supp. Fig. 2A-B). The TR-57-dependent decrease in some of these proteins was confirmed to occur in wildtype (WT) but not ClpP KO TNBC cells by immunoblotting (Supp. Fig. 2C-D). Thus, these results suggested that ClpP agonists affected both pro- and anti-apoptotic protein expression in the TNBC cell lines tested.

### TR-57 Induces Cell Cycle Arrest in Breast Cancer Models

**Figure 3:**
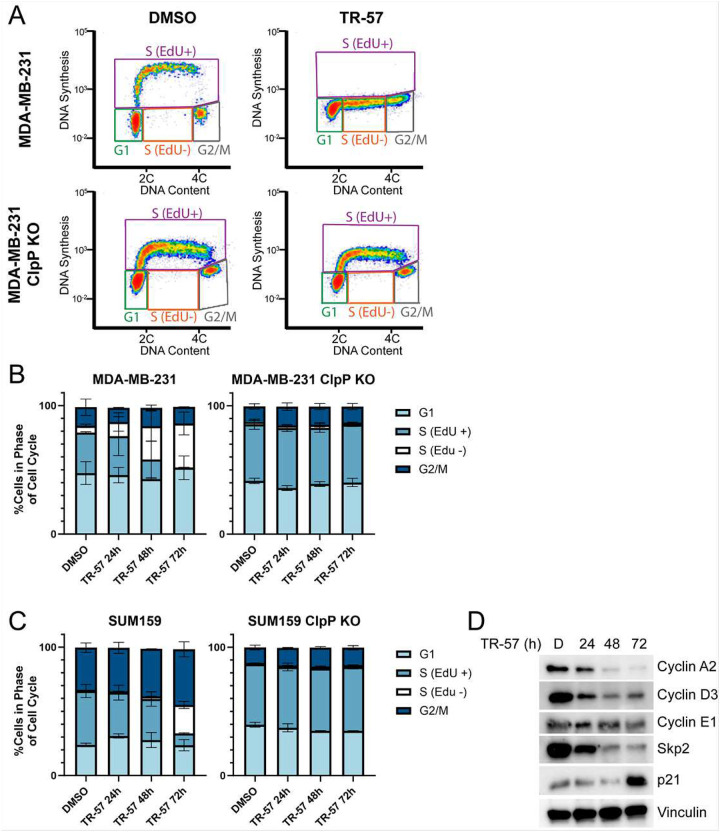
ClpP activation induces an S-phase arrest A) MDA-MB-231 and ClpP KO cells were treated with 0.1% DMSO or 150nM TR-57 for 72h. After a 30 min EdU pulse, cells were harvested and analyzed via flow cytometry. Representative images indicate populations of cells in G1, S with EdU incorporation (EdU+), S without EdU incorporation (EdU-) or G2/M. B-C) Graphed percentages of MDA-MB-231 and SUM159 cells in G1, S EdU+, S EdU-, or G2/M, N=2. D) Representative immunoblots of MDA-MB-231 cells treated with DMSO or TR-57 (24–72h) N=3. All error bars are representative of the standard deviation (SD).

To investigate the effects of ClpP agonists on cell cycle progression, flow cytometry was performed on TNBC cells exposed to TR-57. MDA-MB-231 and SUM159 cells were incubated with TR-57 for 24, 48, or 72h and analyzed as described in Methods. As shown in Fig. 3A, we observed a profound inhibition of cell cycle progression in the WT, but not the ClpP KO cells. Analysis of the flow data demonstrated a strong decrease in EdU staining in S-phase from both MDA-MB-231 and SUM159 cell lines at 72h, indicating that TR-57 was inducing an S-phase arrest (Fig. 3B, C and Supp. Fig. 3A, B). The absence of EdU staining suggested that ClpP activation was inducing replication stress, wherein DNA replication is stalled and EdU cannot be incorporated (61,62). To further characterize the effects of ClpP agonists on cell cycle arrest, we measured the expression of proteins that regulate cell cycle progression. A decrease in cyclins A2, D3, E1, and S-phase kinase associated protein 2 (Skp2) were observed after TR-57 treatment (Fig. 3D). By contrast, p21 expression markedly increased after 72h. Decreased expression of p21 is associated with S-phase arrest, but p21 expression is known to increase during senescence (63). Similar decreases in cyclin A2, D3, E1, and Skp2 protein expression were observed with TR-107 and ONC201 treatment at 72h, indicating this S-phase arrest is consistent across ClpP agonists (Supp Fig. 3C). Taken together this data provides strong evidence supporting an S-phase arrest.

### ClpP agonist treatment induces DNA damage and a DNA damage response

**Figure 4:**
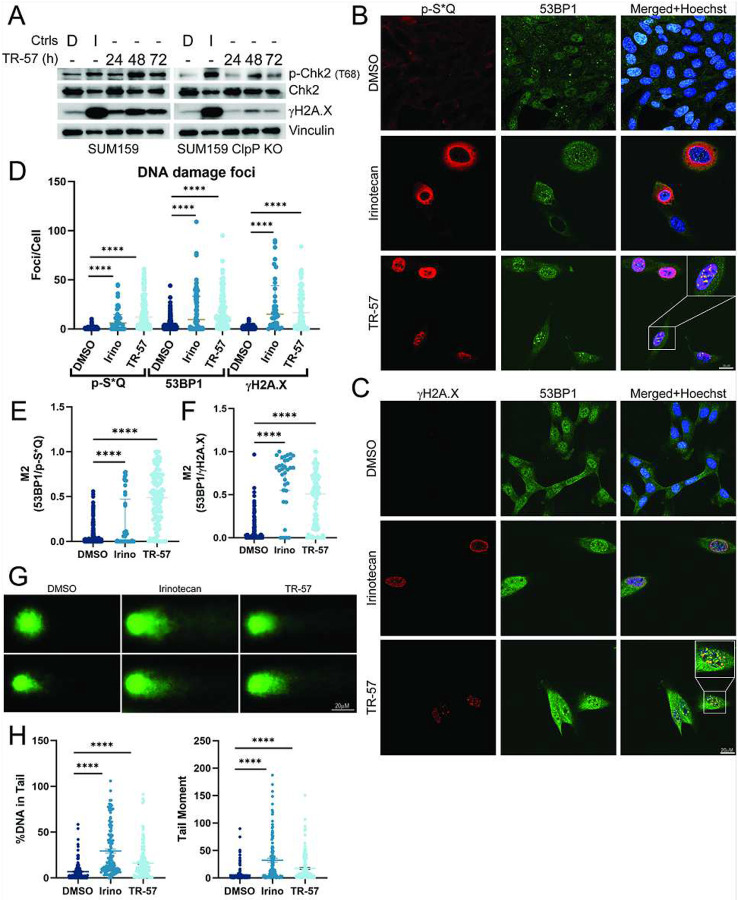
ClpP activation induces DNA damage and the DNA damage response A) Representative immunoblotting images of DNA damage markers from SUM159 and ClpP KO cells treated with 0.1% DMSO for 72h (D), 2μM irinotecan for 48hr (I), or 150nM TR-57 for indicated times, N=3. B) Representative immunofluorescence images of SUM159 cells treated with DMSO, irinotecan (Irino), or TR-57 for 72 h. DNA damage signaling was detected with 53BP1 and p-S*Q foci C) Representative images of γH2A.X and 53BP1 foci after 72h treatment with DMSO, Irino, or TR-57 in SUM159 cells. D) Quantification of p-S*Q substrate, 53BP1, or γH2A.X foci per cell in each treatment condition described in B and C. E-F) Mander’s Coefficient of probability that 53BP1 signal overlaps with p-S*Q or γH2A.X respectively per cell. G) Representative images of DNA from comet assay. H) Quantification of comet assay results. %DNA in Tail and Tail moment were quantified using OpenComet, N=3. All error bars are representative of the standard deviation (SD).

Our previous phosphoproteomics data indicated that TR-57 induced a DNA damage response based on increased phosphorylation of multiple substrates on phospho-S*Q (p-S*Q) sites which are commonly phosphorylated by activated Ataxia-Telangiectasia Mutated (ATM) and Ataxia-Telangiectasia and Rad3-related (ATR) proteins (31). Since DNA damage often precedes the development of senescence and S-phase arrest, we examined the effects of TR-57 on well-established markers of the DNA damage response (DDR). This included 53BP1, γH2A.X, ATM/ATR, and Chk1/2. Western blotting confirmed an increase in Chk2 and H2A.X phosphorylation after TR-57 treatment of SUM159 cells. These results were observed in WT but not ClpP KO cells, whereas they were increased in both cell models with the positive control irinotecan (Fig. 4A). Similar results were observed with MDA-MB-231 cells (Supp. Fig. 4A) and with ONC201 and TR-107 (Supp. Fig. 4B). Moreover, TR-57 and ONC201 increased the phosphorylation of ATM in parallel with increased phosphorylation of Chk2, the effects of which were blocked with the ATM inhibitor KU-60019. However, neither TR-57 nor ONC201 induced Chk1 phosphorylation (Supp. Fig. 4C-D).

Next the influence of TR-57 on the DDR was further investigated by immunofluorescence microscopy. We observed colocalization of 53BP1 with γH2A.X or the p-S*Q binding motif for ATM and ATR substrates after incubation of SUM159 cells with TR-57 or irinotecan (Fig. 4B–C). Quantification of the images confirmed a significant increase in p-S*Q, 53BP1, and γH2A.X foci after TR-57 treatment compared to DMSO (Fig. 4D). The Mander’s coefficient of colocalization for 53BP1 signal overlapping with p-S*Q or γH2A.X were both significantly increased (Fig. 4E–F). Colocalization of 53BP1 and γH2A.X demonstrated a strong correlation (higher M2 value) between 53BP1 location and γH2A.X location, suggesting DNA damage was occurring at these foci (Fig. 4F).

To validate that ClpP activation induces DNA damage, we performed an alkaline comet assay. This assay identifies double- and single-strand breaks in nuclear DNA (Fig. 4G). As shown in Fig. 4H, the alkaline comet assay showed increased signs of DNA damage after TR-57 incubation with SUM159 cells, confirming that TR-57 induces DNA damage in TNBC cells.

### ClpP agonist induced senescence is not reversible

**Figure 5:**
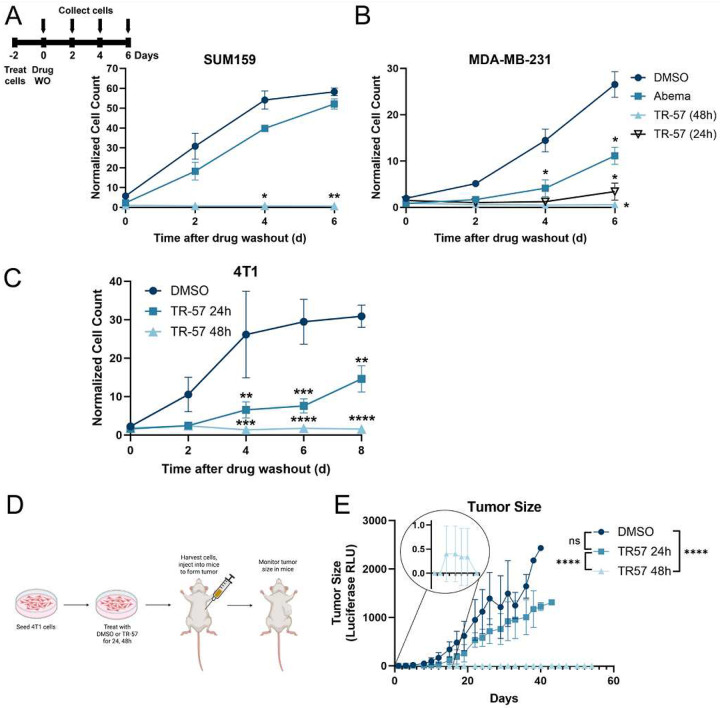
TR-57-induced senescence is irreversible *in vitro* and *in vivo* A-B) SUM159 and MDA-MB-231 cells were treated with 0.1% DMSO (48h), 150nM TR-57 (24–48h) or 500nM abemaciclib (48h), then drugs were washed out and cell count was monitored for 6 days after drug washout, N=3. C) 4T1-Luc cells were treated with TR-57 for 24 or 48h before drug washout. Cell count was measured for 6 days after drug washout, N=3. D.) Scheme for experimental set up in mouse study. 4T1-Luc cells were treated with 0.1% DMSO (48h) or 150nM TR-57 (24 or 48h). The cells were collected and 500 000 cells injected into each mouse. E.) Tumor formation was monitored by relative luminescence units (RLU) twice per week after injection of 4T1-Luc cells, N=8 mice per condition. All error bars are representative of the standard deviation (SD).

To determine if ClpP-agonist treated TNBC cells were truly senescent, we performed TR-57 treatments followed by drug washout and monitored cell proliferation. SUM159 or MDA-MB-231 cells were incubated with DMSO or TR-57 for 24 or 48h at which time the media was replaced with drug-free media (washout) and cells allowed to proliferate. While DMSO-treated cells grew rapidly as expected, cells treated with TR-57 for 24h regained slow growth after washout. By comparison, after 48h TR-57 incubation and washout, there was no increase in growth even enough the cells remained viable 6 days after washout (Fig. 5A–B). By contrast, in washout experiments following treatment with the CDK4/6 inhibitor abemaciclib (48h), we observed rapid recovery of cell proliferation almost immediately after drug washout, especially in the SUM159 cells (Fig. 5A–B). Both SUM159 and MDA-MB-231 ClpP KO cells showed no significant changes between DMSO and TR-57-treated growth after drug washout, but abemaciclib growth curves were consistent with the WT abemaciclib treatments as expected (Supp Fig. 5A-B). Finally, the mouse TNBC cell line, 4T1-Luc, was tested and similar results obtained as in the human TNBC cells. Both TR-57 (24h and 48h) treatments were significantly different from DMSO growth following 4 days of washout, but the TR-57 (48h) treated cells never grew past the initial seeding density (Fig. 5C).

To investigate the underlying mechanism for the sustained senescence induced by TR-57, we blotted for the cell cycle regulators Rb and Myc. Both TR-57 and abemaciclib strongly downregulated Rb phosphorylation after 72h treatments. Following abemaciclib washout (2, 4, 6 days), p-Rb and Myc expression rapidly returned to the pretreatment levels whereas neither Rb, p-Rb or Myc protein levels recovered following TR-57 washout. Instead, these proteins all remained significantly decreased, suggesting a more permanent effect (Supp Fig. 5C). Notably, the absence of TUFM protein indicated mitochondrial function remained impaired even after 6 days of drug washout (Supp. Fig. 5C). Together, this data suggests that TR-57-induced a sustained proliferation arrest after the drug was removed compared to abemaciclib where after drug washout, proliferation returned.

### TR-57 induces stable cell senescence in a cell-line derived xenograft model

To further determine if the TR-57-induced senescence was truly irreversible, we treated 4T1-Luc cells with TR-57 for 24 or 48h and performed drug washout as described in Methods. Viable cells from each treatment condition were injected into the mammary fat pads of BALB/c mice and tumor formation monitored by luciferase activity (Fig. 5D). As expected, the control (DMSO) treated cells rapidly grew tumors. TR-57 (24h) treated cells lagged initially but also showed an increase in tumor growth over time. By contrast, 4T1-Luc cells exposed to TR-57 (48h) before washout, did not show tumor growth at any time during this experiment, even though cells were detectable as demonstrated by inset (Fig. 5E). No significant changes in the mouse weight were observed for the duration of the experiment between groups (Supp. Fig. 5D). 4T1-Luc response to ClpP activation was further demonstrated by Western blots that confirmed reduction of Myc and p-Rb expression, similar to changes observed in the human TNBC cell lines (Supp. Fig 5E). This data suggested that once senescence was established (48h, TR-57 treatment), the senescent phenotype was not reversible *in vivo*.

### Venetoclax synergizes with TR-57 to induce senolysis

**Figure 6:**
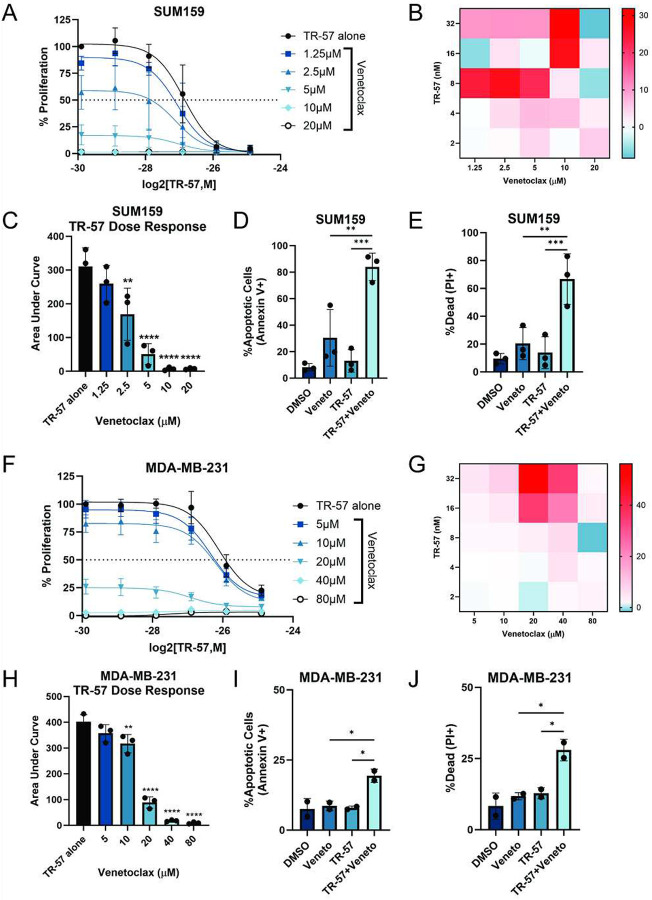
Venetoclax synergizes with TR-57 to induce cell death in TNBC cells A) Dose-response curve of SUM159 cells treated with increasing concentrations of TR-57 titrated with venetoclax, N=3. B) Heat map of HSA synergy scores for graphed combinations of TR-57 and venetoclax in A. C) Area under the curve (AUC) of A. D-E) Flow cytometry was performed on SUM159 cells treated with 0.1% DMSO, 10μM venetoclax (Veneto), 25nM TR-57 or both for 72h. Percent apoptotic and dead cells are graphed respectively, N=3. F) Dose-response curve of MDA-MB-231 cells treated with increasing concentrations of TR-57 titrated with venetoclax, N=3. G) Heat map of HSA synergy scores for graphed combinations of TR-57 and venetoclax in F. H) Area under the curve (AUC) of F. I-J) Flow cytometry was performed on MDA-MB-231 cells treated with DMSO, 20μM venetoclax (Veneto), 25nM TR-57 or both for 72h. Percent apoptotic and dead cells are graphed respectively, N=3. All error bars are representative of the standard deviation (SD).

Because our data showed that TR-57-treated TNBC cells underwent senescence and not apoptosis (Fig. 1), we investigated whether addition of a senolytic would increase cell death. Venetoclax is a Bcl-2 family inhibitor and established senolytic (64–67). The combination of TR-57 and venetoclax resulted in significantly enhanced inhibition of SUM159 and MDA-MB-231 cell growth after 72h treatments (Fig. 6A, F). Synergy between TR-57 and venetoclax was further confirmed by viability analysis based on the highest single agent (HSA) model (Fig. 6B, G). HSA scores greater than 10 indicate a strong chance of synergy. With the largest scores of 32 and 56 for SUM159 and MDA-MB-231 cells respectively, TR-57 and venetoclax demonstrated a synergistic effect on TNBC cell viability. Furthermore, the area under the curve (AUC) indicates a strong combination effect with high doses of venetoclax with TR-57 (Fig. 6C, H). Importantly, this combination resulted in a significant increase in cell death compared to TR-57 or venetoclax alone as measured by annexin V positive staining (Apoptotic cells) (Fig. 6D, I) or propidium iodide (PI) staining (Dead cells) (Fig. 6E, J) in both TNBC cell lines (Supp. Fig. 6A-B). To demonstrate that this synergistic effect was not solely dependent on TR-57, we tested TR-107 and venetoclax and found this combination also significantly increased the percent of apoptotic and dead cells compared to either drug alone (Supp. Fig. 6C).

**Figure 7:**
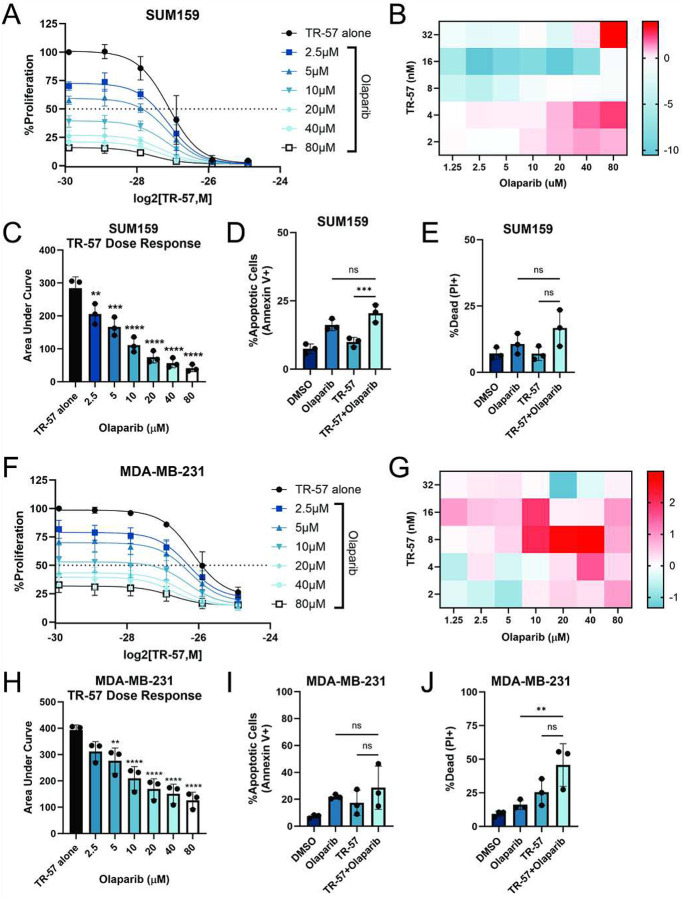
Olaparib and TR-57 co-treatment produces additive cell death effects in TNBC cells A) Dose-response curve of SUM159 cells treated with increasing concentrations of TR-57 titrated with olaparib, N=3. B) Heat map of HSA synergy scores for graphed combinations of TR-57 and olaparib in A. C) Area under the curve (AUC) of A. D-E) Flow cytometry was performed on SUM159 cells treated with 0.1% DMSO, 20μM olaparib, 25nM TR-57 or both for 72h. Percent apoptotic and dead cells are graphed respectively, N=3. F) Dose-response curve of MDA-MB-231 cells treated with increasing concentrations of TR-57 titrated with olaparib, N=3. G) Heat map of HSA synergy scores for graphed combinations of TR-57 and olaparib in F. H) Area under the curve (AUC) of F. I-J) Flow cytometry was performed on MDA-MB-231 cells treated with DMSO, 20μM olaparib, 25nM TR-57 or both for 72h. Percent apoptotic and dead cells are graphed respectively, N=3. All error bars are representative of the standard deviation (SD).

Given the observed increase in the DNA damage response after TR-57 treatment, we examined if co-targeting DNA repair would induce cell death. Specifically, we tested the PARP inhibitor, olaparib in combination with TR-57. Adding increasing concentrations of olaparib and TR-57 demonstrated dose-dependent inhibition of growth of SUM159 and MDA-MB-231 cells after 72h (Fig. 7A, F). Applying synergy calculations (HSA) determined that the highest synergy score was 4 and 3 in the SUM159 and MDA-MB-231 cells respectively. HSA synergy scores between 0 and 10 generally indicate an additive effect, suggesting that TR-57 and olaparib are not synergistic, but do have potential beneficial effects as co-treatments (Fig. 7B, G). The significant decrease in AUC with increasing olaparib concentrations supports evidence of an additive effect (Fig. 7C, H). Measuring cell death by flow cytometry demonstrated an increase in cell death in response to this combination that further suggested additive effects in both TNBC cell lines (Fig. 7D–E, 7I–J, Supp Fig. 6B).

## Discussion:

Represented by ONC201 and the TR compounds, small molecule ClpP activators (ClpP agonists) have generated considerable interest as anti-cancer agents with a novel mechanism of action. The fact that ONC201 (Dordaviprone) is now an FDA-approved monotherapy, suggests enormous promise for this novel modality of cancer treatment. These compounds show broad activity against multiple cancer types with increased cell death (apoptosis) most commonly observed (33,34,68–70). In this study we demonstrate that senescence is the dominant outcome in TNBC models. We confirmed this by examining a multitude of well-established senescence markers and demonstrating the permanence of this response, both in cell cultures and an animal model. Before TR-57 induces senescence in TNBC cell lines, DNA damage and S-phase arrest were observed, but we were unable to demonstrate that DNA damage or S-phase arrest was the cause of senescence in these cells. However, taking advantage of the senescent response, we show that ClpP agonists can be combined with the known senolytic venetoclax to increase cell death.

TR-57 was shown to induce senescence by significantly increasing β-gal activity and SASP cytokines and decreasing Lamin B1 expression and proliferation markers (p-Rb and Myc). A 30-day TR-57 treatment showed no increase in, or loss of cell count, further demonstrating the senescent phenotype. Analysis of our proteomics data characterized the properties of ClpP agonist-induced senescence. This identified well-established markers of senescence (i.e. GDF15) and events known to precede senescence such as increased DNA damage, cell cycle arrest, and cytokine production. Interestingly we also observed protein markers that may be unique to the action of ClpP agonists including upregulation of multiple amino acid transporters and metabolic enzymes. Whether or not these proteins contribute to the increased secretion or altered metabolism of senescent cells remains to be established.

An increase in DNA damage following ClpP activation is well supported by our evidence (γH2A.X and 53BP1 foci, comet formation, ATM/CHK2 activation), and the timing of DNA damage correlates with the induction of senescence (Fig 4A, Supp. Fig 4E.) However, the origin of the DNA damage response remains unclear. Unexpectedly, ATR and Chk1 phosphorylation were not observed after ClpP activation, unlike that seen with ATM and Chk2 (Supp. Fig. 4C). A possible explanation is our finding that TR-57 induces S-phase arrest. ATR activity is essential for protecting replication forks during S-phase, and blocking ATR leads to replication stress (62). The lack of ATR/Chk1 activation combined with an S-phase arrest suggests ClpP activation is inducing replication stress. The cause of DNA damage and S-phase arrest was not determined. However, one possibility is that increased ROS is responsible, although this was not directly measured in this study. Moreover, the antioxidant, N-acetylcysteine failed to block γH2A.X phosphorylation, a strong indicator of DNA damage (Supp. Fig. 7A). An alternative hypothesis is that the DNA damage results from nucleotide depletion. Because ClpP activation impairs pyrimidine biosynthesis and limits the availability of nitrogenous bases (31), we tested whether the observed DNA damage was caused by nucleotide depletion. However, supplementation of MDA-MB-231 cells with pyrimidines and/or purines during TR-57 treatment did not prevent the DNA damage response, suggesting nucleotide depletion was not the cause of TR-57-induced DNA damage (Supp. Fig. 7B). Studies, including our own, have correlated mitochondrial dysfunction and nuclear DNA damage (71–73), but how mitochondrial dysfunction leads to increased nuclear DNA damage is still unknown.

We observed evidence for S-phase arrest in response to ClpP agonists contrary to previous reports identifying a G1 arrest (20,74). Those studies, conducted in lung and colorectal cancers, relied only on DAPI or PI staining, which can make it difficult to distinguish S-phase arrest without the added resolution of EdU incorporation. Thus, it is possible that the S-phase arrest went undetected in those analyses. Alternatively, these differences may reflect cancer type–specific effects of ClpP activation. The observed S-phase arrest was characterized by an inhibition of EdU incorporation, consistent with increased DNA replication stress and arrest. Decreases in cyclin A2, cyclin D3, and Skp2 further indicate a deregulated S-phase. These responses were ClpP-dependent and differed from that observed with abemaciclib (G1 arrest). Thus, the induction of cell cycle arrest by ClpP agonists appears to be unique and may explain the irreversibility observed with these agents. Abemaciclib-induced growth inhibition was rapidly reversed following drug washout, but growth inhibition by ClpP activation was not. This coincided with a drastic decline in Myc protein that was not observed following abemaciclib treatment. Importantly, the loss of Myc was not readily reversed upon ClpP agonist washout, suggesting that Myc downregulation may play an important role in the sustained inhibition of cell proliferation and development of senescence. Our *in vivo* model further demonstrates the irreversibility of TR-57-induced senescence, though further studies are needed to determine how ClpP activation causes a permanent proliferation arrest.

Therapy-induced senescence is relevant in the context of cancer (42) and many chemotherapeutics induce cancer cell senescence including cisplatin, irinotecan, bleomycin, and doxorubicin (42,75–78). Ionizing radiation increases DNA damage and senescence and is one of the most commonly used methods of inducing senescence in models of aging (79,80). ClpP agonists induce senescence in TNBC cells, whereas apoptosis is more common in other cancers, such as H3K27 mutant gliomas targeted by ONC201. TR-57 did not significantly increase apoptosis in TNBC (Fig. 6D, I), suggesting these cells are uniquely resistant to ClpP-induced apoptosis. The apoptotic arrays (Supp. Fig 2) did not reveal any specific vulnerabilities of TR-57-induced senescent TNBC cells. Instead, we sought to induce cell death in TNBC with co-treatment of ClpP agonists and a well-established senolytic (venetoclax). As shown here and earlier, venetoclax significantly increased cell death in TNBC and leukemia cells (67). Furthermore, synergy was observed between TR-57 and venetoclax as observed by HSA synergy scores. These studies suggest that successful application of ClpP agonists may depend on the pro- and anti-apoptotic properties of the cancer cell types in question.

Combination experiments with TR-57 and olaparib had additive effects, suggesting that increasing DNA damage is beneficial to the mechanism of action of ClpP agonists. However, the relationship is not synergistic, and this observation was confirmed by testing other DNA damaging agents (doxorubicin, 5-FU, irinotecan). There was a distinct additive effect to treating TR-57 with DNA damaging agents, but the effects are not synergistic (Supp. Fig. 7D). Ultimately, there may be more therapeutic rationale for combination treatments targeting anti-apoptotic proteins than targeting DNA and DNA repair with ClpP agonists in TNBC.

In summary, we describe the effects of novel small molecule activators of ClpP on TNBC cells and the events preceding and/or contributing to the development of senescence. Compared to other cancer models where ClpP agonists induce apoptosis or other forms of cell death (i.e., ferroptosis), senescence predominates in the TNBC models tested here. As such, these studies may have important implications for potential treatments of this specific type of cancer with ClpP agonists.

ClpP agonists represent a novel class of anti-cancer agents with promising results in many cancers, exemplified by the FDA-approved ONC201. In TNBC models, ClpP agonists predominantly induce senescence. TR-57-induced senescence is preceded by DNA damage and S-phase arrest, and the associated downregulation of Myc may contribute to the irreversibility of this response. Importantly, TNBC cells are resistant to apoptosis, but combination with the senolytic venetoclax produces significant synergistic cell death, highlighting a promising therapeutic strategy. These findings provide mechanistic insight into ClpP agonist action in TNBC cells and identify a combinatorial approach that may offer new therapeutic options for breast cancer patients with few targeted treatments available.

## Supplementary Material

Supplementary Figure Legends:

Supplementary Figure 1: ClpP Agonists Induce Senescence in TNBC cells

A) Representative images of quantified β-gal staining data from Figure 1B. B) Representative images of MDA-MB-231 cells from Fig 1C. C) Representative images of SUM-159 cells from Fig 1D. D) SUM159 cells were treated with abemaciclib (48h) or TR-57 for 24–72h and immunoblotting was performed to measure protein expression of senescence markers. Representative images of N=2 E) Immunoblot of increasing concentrations of TR-57 and GDF15 expression in SUM159 cells. N=2 F) Comparison of dose-response curves from 3 or 30 days of TR-57 treatment. IC_50_ values were plotted for both cell lines in graph as shown on the right. Representative curves from N=3 G) Immunoblot of ONC201, TR-57, TR-107, abemaciclib, and irinotecan (2μM) demonstrating changes in protein expression of senescence markers. Representative images of N=2.

Supplementary Figure 2: ClpP Agonists Deplete Pro- and Anti-Apoptotic Proteins

A) SUM159 and MDA-MB-231 cells were treated with 0.1% DMSO or 150nM TR-57 for 48h and an immunoblotting array was utilized on cell lysates to visualize proteins known to regulate apoptosis. B) Quantification of SUM159 array. C-D) SUM159 cells and MDA-MB-231 cells respectively were treated with 0.1% DMSO, 100nM staurosporine (S) for 24h, 500nM abemaciclib (A) for 48h, or 150nM TR-57 for 48h. Validation of changes in protein expression from array was performed by immunoblotting. Representative images of N=2–3.

Supplementary Figure 3: ClpP Agonists Induce S-phase Arrest

A) Gating scheme for cell cycle analysis in Figure 3. B) Representative plots of SUM159, MDA-MB-231 and respective ClpP KO cells treated with 0.1% DMSO or 150nM TR-57 for 24–72h from Figure 3B–C. C) MDA-MB-231 cells were treated with 150nM TR-57, 10μM ONC201, or 100nM TR-107 for 72h. Immunoblotting was performed on cell-cycle proteins. Representative images of N=2

Supplementary Figure 4: ClpP Agonists Induce DNA Damage in TNBC cells

A) MDA-MB-231 and ClpP KO cells were treated with 0.1% DMSO (D) for 48h, 2μM irinotecan (I) for 48h, and 150nM TR-57 for 24–72h. Immunoblotting was performed to detect DNA damage markers. Representative images of N=2. B). MDA-MB-231 cells were treated with 150nM TR-57, 10μM ONC201, 100nM TR-107, 500nM abemaciclib, or 2μM irinotecan for 72h. Immunoblotting was performed on DNA damage markers. Representative images of N=2. C-D) SUM159 cells were treated with the indicated drugs for 48h and immunoblotted as shown. A positive control, indicated by (+ Ctrl), consisted of 250μM H_2_0_2_ for 30 min for all samples except p-Chk1 and Chk1 which was 50μM etoposide for 2h. N=2. E) SUM159 cells were untreated (time 0) or treated with 50nM TR-57 for the indicated times and immunoblotted as shown. A positive control, indicated by (+), consisted of 100μM H_2_0_2_ for 30 min for all samples except p-ATR, ATR, and p-Chk1 which was 50μM etoposide for 2 h.

Supplementary Figure 5: TR-57-induced senescence is not reversible in *in vitro* and *in vivo* TNBC models

A-B) SUM159 or MDA-MB-231 ClpP KO cells respectively were treated with 0.1% DMSO, 500nM abemaciclib, or 150nM TR-57 for 48h before drug washout. Cell count was monitored for 6 days after drug washout. N=3. C) MDA-MB-231 cells were treated with above drugs for 72h before drug washout. Cells were collected every 2 days after washout for up to 6 days. Immunoblotting was performed on proliferation markers and TUFM. Representative images of N=2. D) Weights of mice from experiment described in Figure 5D–E. E) 4T1-Luc cells were treated with 0.1% DMSO or 150nM TR-57 for 24–72h and immunoblotting was performed on proliferation markers and TUFM to demonstrate ClpP activation. Representative images of N=2.

Supplementary Figure 6: Venetoclax or Olaparib Co-Treatment with ClpP Agonists Increase Cell Death in TNBC Cells

A-B) Example gating scheme and representative plots for SUM159 and MDA-MB-231 cells respectively. C) SUM159 cells were treated with 0.1% DMSO, 10μM venetoclax (Veneto), or 25nM TR-107 for 72h. Annexin V/PI staining was quantified to determine %Apoptotic (Annexin V+) or %Dead (PI+). N=3.

Supplementary Figure 7: Effects of TR-57 are not reversed by NAC or nucleotide supplementation while doxorubicin co-treatment induces additive effects

A) MDA-MB-231 cells were treated with 0.1% DMSO (D), 1μM Rotenone (R), 150nM TR-57 (TR), and/or 5mM N-acetyl cysteine (NAC) for 48h. Immunoblots of γH2A.X were performed to assess changes in DNA damage. Representative images of N=2. B) MDA-MB-231 cells were treated with 0.1% DMSO, 50μM Teriflunomide (TF), 150nM TR-57 (TR), and/or 30μM Uridine, 100μM Cytidine, and 100μM Thymidine (U+C+T), or 100μM Adenosine and 100μM Guanosine (A+G) for 72h. Representative images of N=2. C) SUM159 cells were treated with varying concentrations of TR-57 with doxorubicin titrated in. Cell count and viability were measured after 72h of co-treatment. HSA plot indicates additive effects with co-treatment. Representative of N=2 experiments.

Supplementary Files

This is a list of supplementary files associated with this preprint. Click to download.

- SupplementaryMethodTables.docx
- UncroppedWesternblots.pdf
- SuppFigure7PotentialcausesofDNAdamage.tif
- SuppFigure5WOmousedata.tif
- SuppFigure6TR57VenSynergy.tif
- SuppFigure3SphasearrestFlow.tif
- SuppFigure2ProandAntiApoptoticProteins.tif
- SuppFigure4DNAdamage.tif
- SuppFigure1Senescence.tif

## Data Availability

The proteomics dataset generated and analyzed during the current study are available in the Proteomics Identification Database (PRIDE) repository under project identifier PXD067842. The remaining datasets generated and/or analyzed during the current study are available from the corresponding author on reasonable request.
